# Long-Term Survival in Patients With Low-Risk Cervical Cancer After Simple, Modified, or Radical Hysterectomy

**DOI:** 10.1001/jamanetworkopen.2025.10717

**Published:** 2025-05-15

**Authors:** Christopher M. Tarney, Chunqiao Tian, Leslie M. Randall, S. Ahmed Hussain, Pouya Javadian, Sean P. Cronin, Sara Drayer, John K. Chan, Daniel S. Kapp, Chad A. Hamilton, Charles A. Leath, Doris M. Benbrook, Christina R. Washington, Kathleen N. Moore, Nicholas W. Bateman, Thomas P. Conrads, Neil T. Phippen, G. Larry Maxwell, Kathleen M. Darcy

**Affiliations:** 1 Gynecologic Cancer Center of Excellence, Department of Gynecologic Surgery and Obstetrics, Uniformed Services University of the Health Sciences, Walter Reed National Military Medical Center, Bethesda, Maryland; 2 Murtha Cancer Center Research Program, Department of Surgery, Uniformed Services University of the Health Sciences, Bethesda, Maryland; 3 The Henry M. Jackson Foundation for the Advancement of Military Medicine Inc, Bethesda, Maryland; 4 Mid-Atlantic Gynecologic Oncology and Pelvic Surgery Associates, Inc, Inova Shar Cancer Institute, Falls Church, Virginia; 5 Women’s Health Integrated Research Center, Inova Women’s Service Line, Inova Health System, Falls Church, Virginia; 6 Palo Alto Medical Foundation, California Pacific Medical Center, Sutter Health, San Francisco, California; 7 Department of Radiation Oncology, Stanford University School of Medicine, Stanford, California; 8 Gynecologic Oncology Section, Women’s Services, The Ochsner Cancer Institute, Ochsner Health, New Orleans, Louisiana; 9 Division of Gynecologic Oncology, University of Alabama at Birmingham, O’Neal Comprehensive Cancer Center, Birmingham; 10 Gynecologic Oncology Division, Stephenson Cancer Center, Oklahoma University Health Sciences Center, Oklahoma City; 11 Advent Health Medical Group, Gynecologic Oncology at Porter, Denver, Colorado

## Abstract

**Importance:**

Three-year pelvic recurrence rate in women with low-risk cervical carcinoma was not inferior following simple hysterectomy (SH) vs modified radical hysterectomy (MRH) or radical hysterectomy (RH) in the Simple Hysterectomy and Pelvic Node Assessment randomized clinical trial, but the survival analysis of the trial was underpowered.

**Objective:**

To evaluate long-term survival in low-risk cervical carcinoma following SH vs MRH or RH.

**Design, Setting, and Participants:**

This cohort study included women undergoing SH, MRH or RH in US Commission on Cancer–accredited facilities participating in the National Cancer Database who received a diagnosis between January 2010 and December 2017 of International Federation of Gynecology and Obstetrics 2009 stage IA2 or IB1 squamous cell carcinoma, adenocarcinoma, or adenosquamous carcinoma of the cervix (≤2 cm) and clinically negative lymph nodes.

**Exposure:**

SH, MRH, or RH following diagnosis of stage IA2 or IB1 squamous cell carcinoma, adenocarcinoma, or adenosquamous carcinoma of the cervix.

**Main Outcomes and Measures:**

Survival was the primary end point, evaluated with and without propensity score balancing. Survival rates, survival distributions, adjusted hazard ratio (aHR) of death, and restricted mean survival times (RMST) were analyzed as of September 2024. Two multivariable models were fitted. Model 1 included the hysterectomy type and 9 baseline factors (age, comorbidity score, race and ethnicity, insurance status, treatment facility, stage, histologic subtype, tumor grade, and surgical approach). Model 2 included the model 1 variables plus 4 additional clinical factors (surgical margin, LVSI, pathologic LN metastasis, and adjuvant treatment).

**Results:**

This cohort study evaluated 2636 women (mean [SD] age, 45.4 [11.4] years; median [IQR] follow-up, 85 [64-110] months), including 982 with SH, 300 with MRH, 927 with traditional RH, and 427 with unspecified MRH or RH. Survival was similar following SH vs MRH or RH (7 year survival rate, 93.9%; 95% CI, 91.9%-95.4% vs 95.3%; 95% CI, 94.0%-96.3%%; *P* = .07) and SH vs MRH vs RH (7 year survival rate, 93.9%; 95% CI, 91.9%-95.4% vs 94.2%; 95% CI, 90.1%-96.7% vs 95.4%; 95% CI, 93.6%-96.6%; *P* = .15). Risk of death following either SH vs MRH or RH, SH vs RH, or MRH vs RH remained similar after adjusting for baseline covariates alone or baseline covariates plus clinical factors. Survival remained similar within subsets by age, comorbidity score, race and ethnicity, facility type, stage, histologic subtype, tumor grade, surgical approach, and year of diagnosis. Adjusted survival remained similar in patients with SH vs MRH or RH after propensity score balancing for baseline covariates (aHR, 1.19; 95% CI, 0.86-1.65; *P* = .31) with similar 3-year (98.3%; 95% CI, 97.2%-99.0% vs 97.6%; 95% CI, 96.6%-98.2%), 5-year (95.9%; 95% CI, 94.3%-97.1% vs 96.5%; 95% CI, 95.5%-97.3%), 7-year (94.5%; 95% CI, 92.5%-95.9% vs 95.1%; 95% CI, 93.7%-96.1%), and 10-year (89.8%; 95% CI, 86.3%-92.5% vs 91.7%; 95% CI, 89.4%-93.4%) survival rates. Sensitivity analysis for patients who received a diagnosis between 2010 and 2013 documented similar 10-year RMST following SH vs MRH or RH, SH vs RH, SH vs MRH, and MRH vs RH.

**Conclusions and Relevance:**

In this cohort study, long-term survival was similar following SH vs MRH or RH, supporting the use of SH in select patients with low-risk early-stage cervical carcinoma.

## Key Points

**Question:**

Do long-term survival and postoperative outcomes vary following simple hysterectomy (SH) vs modified radical hysterectomy (MRH) or radical hysterectomy (RH) in patients with low-risk early-stage cervical carcinoma?

**Findings:**

In this cohort study of 2636 patients with International Federation of Gynecology and Obstetrics 2009 stage IA2 or IB1 cervical carcinoma, patients had similar 3-, 5-, 7-, and 10-year survival and consistent rates of positive margin, lymphovascular space invasion, pathological node metastasis, 30-day hospital readmission, and adjuvant treatment following SH vs MRH or RH.

**Meaning:**

This cohort study found similar long-term survival and postoperative metrics in patients undergoing SH vs as MRH or RH, supporting the use of less radical surgery in select patients with low-risk, early-stage cervical carcinoma.

## Teaser

This cohort study assesses long-term survival and postoperative metrics in patients with early-stage cervical carcinoma who underwent simple, modified radical, or radical hysterectomy.

## Introduction

Cervical carcinoma is the fourth most common malignant neoplasm in women globally.^1^ Most patients with cervical carcinoma receive a diagnosis at an early stage due to availability of screening, vaccination, and treatment services.^2,3^ Historically, the National Comprehensive Cancer Network recommended radical hysterectomy (RH) with bilateral pelvic lymphadenectomy for patients not desiring future fertility.^4^ RH entails removing the cervix, uterus, parametria, and upper one-quarter to one-third of the vagina with overall survival rates of up to 90%.^4^ Nevertheless, RH is associated with a 10% to 15% risk for postoperative complications,^5,6^ including hemorrhage, postoperative bowel dysfunction, ureteral fistula, voiding dysfunction, sexual dysfunction, and worse quality of life.^7,8,9,10,11^

Over the past decade, conservative surgical options have been successfully introduced for the treatment of different gynecologic malignant neoplasms, prompting studies to determine if simple hysterectomy (SH), which spares the parametria and upper vagina, can be used for low-risk early-stage cervical carcinoma to mitigate RH-associated morbidities. Retrospective data suggest the probability of parametrial disease is less than 1% for International Federation of Gynecology and Obstetrics (FIGO) 2009 stage IB1 cervical cancer, which is considered to be low-risk, defined as lesions of 2 cm or less, negative nodes, absent lymphovascular space invasion (LVSI), and depth of stromal invasion less than 10 mm.^12,13,14^ A multicenter nonrandomized prospective trial by Schmeler et al^15^ evaluated the performance of less radical surgery for low-risk cervical carcinoma but did not include a comparison with RH with nodal assessment. Schmeler et al^15^ reported a 2-year recurrence rate of 3.5% following SH in 100 patients with FIGO 2009 stage IA2 or IB1 cervical squamous cell-carcinoma (SCC) or adenocarcinoma (AC) with tumors less than 2 cm, no LVSI, and stromal invasion less than 10 mm.

Most recently, Plante et al^16^ published the results of the Simple Hysterectomy and Pelvic Node Assessment (SHAPE) multicenter, randomized, noninferiority trial comparing RH with SH in 700 patients who underwent lymph-node assessment for SCC, AC, or adenosquamous carcinoma (ASC) with low-risk early-stage cervical carcinoma, defined as FIGO 2009 stage IA2 or IB1 tumors with lesions measuring 2 cm or less and stromal invasion less than 10 mm. Although SHAPE^16^ reported comparisons between SH vs RH, patients randomized to RH underwent a type 2 RH or modified radical hysterectomy (MRH); the incidence of pelvic recurrence at 3 years was 2.17% in the RH groups vs 2.52% in the SH group. There were lower urinary complications following SH. The authors concluded that SH was not inferior to RH with respect to 3-year pelvic recurrence rate in patients at low risk. The SHAPE trial was not powered to evaluate survival. With a median follow-up of 4.5 years, there were only 14 deaths reported in the SHAPE trial,^16^ including 7 deaths following SH and 7 deaths following RH. The hazard ratio (HR) for death for SH vs RH was 1.09 (95% CI, 0.38-3.14).^16^ These results supported changes to National Comprehensive Cancer Network guidelines that include conservative surgery as an option in management of patients with low-risk early-stage cervical carcinoma (ie, less than 2cm, no LVSI, negative cone margins, and less than 50% stromal invasion).^17^ There remain questions whether there are differences in short- and long-term survival for patients with low-risk, early-stage cervical carcinoma undergoing conservative surgery.

The primary objective of our observational study is intended to supplement the SHAPE^16^ randomized clinical trial by evaluating short- and long-term survival in patients with low risk, early-stage cervical carcinoma defined using SHAPE criteria following conservative SH vs MRH or RH in the US National Cancer Database (NCDB). Secondary objectives include a pairwise comparisons in risk of death between SH vs RH, SH vs MRH, and MRH vs RH in patients who received a diagnosis between 2010 and 2017, a sensitivity analysis of 10-year survival in a subset of patients with low-risk cervical carcinoma who received a diagnosis between 2010 and 2013, and an examination of postoperative metrics following SH vs MRH or RH. We hypothesized that survival, surgical approach, and postoperative metrics in Commission on Cancer–accredited facilities participating in the NCDB would be similar following SH vs MRH or RH.

## Methods

### Data Sources, Patients, and Study Outcomes

This cohort study was approved by WCG institutional review board with an exemption of informed consent and HIPAA authorization due to the use of deidentified data from the NCDB. The reporting followed the Strengthening the Reporting of Observational Studies in Epidemiology (STROBE) reporting guideline. We evaluated women receiving treatment in US Commission on Cancer–accredited facilities participating in the NCDB who received a diagnosis between 2010 and 2017 of primary FIGO 2009 stage IA2 or IB1 SCC, AC, or ASC of the cervix and underwent SH, MRH, or RH in the NCDB.^18,19^ The primary end point for this study was overall survival, namely the interval from diagnosis to the date of last contact or death from any cause. We also evaluated postoperative positive surgical margin, LVSI, pathologic lymph node (LN) metastasis, hospital readmission within 30 days, and administration of adjuvant treatment.

### Exposure and Control Groups

SH was considered the exposure group, including total hysterectomy with or without removal of tubes and ovaries. MRH or RH was the control group. MRH also incorporated extended hysterectomy. RH included removal of the cervix, uterus, parametria, and upper one-quarter to one-third of the vagina. There were also patients who underwent unspecified MRH or RH.

### Eligibility and Exclusions

All patients underwent SH, MRH, or RH within 60 days from diagnosis with tumor size of 2 cm or less, no LN metastasis on pretreatment clinical evaluation, and pelvic LN dissection. Histological subtypes other than SCC, AC, or ASC were excluded. Exclusion criteria also included multiple primary malignant neoplasms, unspecified timing of hysterectomy, and receipt of neoadjuvant radiotherapyor chemotherapy.

### Covariates and Confounding Variables

The predefined baseline covariates for this study were provided by the NCDB registry, including age at diagnosis, Charlson-Deyo comorbidity score, race and ethnicity, year of diagnosis, insurance status, treatment facility, stage, histologic subtype, tumor grade, and surgical approach. The race and ethnicity variables provided by the NCDB were crossed to generate the race and ethnicity groups, including Asian or Pacific Islander, non-Hispanic Black, Hispanic (including any or missing race), non-Hispanic White, and patients with other race or ethnicity (other includes American Indian, Aleutian, Inuit, or Yupik patients and patients with missing race or ethnicity); race and ethnicity were included in this cohort study given the well documented racial and ethnic differences in disease presentation and outcomes of women with cervical cancer. Surgical approach was classified as minimally invasive surgery (MIS) for robotic-assisted or laparoscopic surgery, open surgery (including both open or MIS converted to open), or other surgery for an unknown or not reported approach. These variables were selected based on their historical clinical and prognostic values for this patient population.

### Statistical Analysis

All statistical analyses were completed in September 2024 using SAS version 9.4 (SAS Institute). Two-sided tests were performed, with statistical significance defined as *P* < .05. No adjustments were made for multiple testing. Comparison of patient and clinical characteristics between SH vs MRH or RH groups was conducted using the *t* test for age as a continuous variable and the *χ^2^* test for the categorical covariates as provided by the NCDB. Follow-up duration was calculated using the reverse Kaplan-Meier method and summarized with the median and IQR. A Cox model stratified by year of diagnosis and corrected for baseline covariates was used to estimate adjusted HRs (aHRs) and 95% CIs for risk of death in SH vs MRH or RH. Two multivariable models were fitted. Model 1 included the hysterectomy type and 9 baseline factors (age, comorbidity score, race and ethnicity, insurance status, treatment facility, stage, histologic subtype, tumor grade, and surgical approach). Model 2 included the model 1 variables plus 4 additional clinical factors (surgical margin, LVSI, pathologic LN metastasis, and adjuvant treatment). Subgroup analyses were performed by extending the multivariable Cox model 1 with an interaction term to explore the consistency of the association of hysterectomy type with survival across subgroups categorized by age, comorbidity score, race and ethnicity, facility type, stage, histologic subtype, tumor grade, and surgical approach.

The association of hysterectomy type with survival was further assessed using propensity score analysis, with inverse probability of treatment weighting applied to balance the clinical covariates following SH vs MRH or RH.^20^ Balance quality between the 2 groups was examined using the standardized mean difference, with a value of less than 10% considered well-balanced.^20^ Adjusted survival in propensity score–balanced patients was estimated using the weighted Kaplan-Meier method following SH vs MRH or RH and compared using a log-rank test. Survival rates with 95% CIs were provided after 3, 5, 7, and 10 years following diagnosis.^20^ The aHRs for risk of death were estimated using a weighted Cox model, with 95% CIs calculated using a robust sandwich variance.^20^ Secondary outcomes were compared in the propensity score–balanced cohort using the weighted *χ^2^* method. In patients who received a diagnosis between 2010 and 2013, for whom the follow-up time was 10 years or more, a sensitivity analysis was performed using restricted mean survival time (RMST), which is defined as the area under the survival curve up to 10 years, allowing a direct comparison of the average survival time between the 2 groups over this follow-up period.^21,22^ An RMST-based linear model was fitted by including the hysterectomy type and 9 baseline covariates, with adjusted difference in 10-year RMST between SH vs MRH vs RH groups calculated using the pseudo-value method.

A post hoc power analysis was also conducted. Based on the number of events observed in this cohort, this study achieved 90% power to detect a 3.4% difference in 7-year survival (ie, 95.0% for MRH or RH vs 91.6% for SH).

## Results

This cohort study evaluated 2636 women who received a diagnosis of low-risk early-stage SCC, AC, or ASC at a mean (SD) age of 45.4 (11.4) years, including 982 who underwent SH and 1654 who underwent MRH or RH. The latter group included 300 patients with MRH, 927 with RH, and 427 with unspecified MRH or RH. Of all participants, 2289 (86.8.%) had stage IB1 disease, and 411 (15.6%) received adjuvant therapy after surgery, including 175 (17.8%) in the SH group and 236 (14.3%) in the MRH or RH group. The eFigure in Supplement 1 displays the patient inclusions and exclusions. Table 1 summarizes the clinical characteristics overall and categorized by type of hysterectomy. Survival was similar following SH vs MRH or RH in the original cohort. The 7-year survival rate was 93.9% (95% CI, 91.9%-95.4%) for the SH group vs 95.3% (95% CI 94.0%-96.3%) for the MRH or RH group overall (P = .07), or 94.2% (95% CI, 90.1%-96.7%) for the MRH group vs 95.4% (95% CI, 93.6%-96.6%) for the RH group (*P* = .15). Patients undergoing SH tended to be older (mean [SD] age, 46.6 [12.2] vs 44.7 [10.9] years; *P* < .001) and more likely than those undergoing MRH or RH to have an MIS approach (656 patients [66.8%] vs 1008 patients [60.9%]; *P* < .001). Modest differences by insurance status, treatment facility, stage, and histologic subtype were present. Median (IQR) follow-up was 85 (64-110) months with 156 observed deaths. Follow-up duration was similar between the 2 groups, with a median (IQR) of 84 (64-109) months in the SH and 85 (63-111) months in the MRH or RH group. Table 1 also presents clinical characteristics, surgical findings and first-line treatments administered in the propensity score–balanced cohort of patients with low-risk, early-stage cervical carcinoma following hysterectomy. Baseline clinical covariates in Table 1 were well balanced with a standardized mean difference less than 1.0%.

**Table 1.** Clinical Characteristics, Surgical Findings, and Treatments for Patients With Low-Risk Cervical Carcinoma Overall and Following Hysterectomy in the National Cancer Database

Clinical characteristics	Cases, No. (%)				Cases, adjusted cohort after propensity score balancing, No. (%)^a^			
	All patients (N = 2636)	SH (n = 982)	MRH or RH (n = 1654)^b^	*P* value^c^	SH (n = 979)	MRH or RH (n = 1652)^b^	Standardized mean difference (%)	*P* value^d^
Age, y								
Mean (SD)	45.4 (11.4)	46.6 (12.2)	44.7 (10.9)	<0.001	45.5 (11.6)	45.5 (11.4)	0.1	NA
<30	134 (5.1)	46 (4.7)	88 (5.3)	NA	49 (5.0)	83 (5.1)	0.3	NA
30-39	788(29.9)	272 (27.7)	516 (31.2)		296 (30.3)	497 (30.1)	0.4	
40-49	849 (32.2)	300 (30.6)	549 (33.2)		308 (31.5)	522 (31.6)	0.3	
50-59	533 (20.2)	207 (21.1)	326 (19.7)		201 (20.5)	338 (20.5)	0.2	
60-69	253 (9.6)	115 (11.7)	138 (8.3)		95 (9.7)	160 (9.7)	0.1	
≥70	79 (3.0)	42 (4.3)	37 (2.2)		30 (3.1)	53 (3.2)	0.5	
Comorbidity score								
0	2316 (87.9)	862 (87.8)	1454 (87.9)	.97	860 (87.9)	1449 (87.7)	0.5	NA
1	266 (10.1)	99 (10.1)	167 (10.1)		100 (10.2)	170 (10.3)	0.4	
≥2	54 (2.1)	21 (2.1)	33 (2.0)		19 (2.0)	33 (2.0)	0.2	
Race and ethnicity^e^								
Asian or Pacific Islander	140 (5.3)	41 (4.2)	99 (6.0)	.22	51 (5.2)	84 (5.1)	0.6	NA
Black	214 (8.1)	86 (8.8)	128 (7.7)		79 (8.0)	134 (8.1)	0.4	
Hispanic	319 (12.1)	117 (11.9)	202 (12.2)		122 (12.5)	204 (12.3)	0.5	
White	1841 (69.8)	687 (70.0)	1154 (69.8)		682 (69.6)	1153 (69.8)	0.3	
Other	122 (4.6)	51 (5.2)	71 (4.3)		45 (4.6)	77 (4.7)	0.4	
Insurance status								
Private	1760 (66.8)	664 (67.6)	1096 (66.3)	.03	656 (67.0)	1104 (66.8)	0.5	NA
Medicare	224 (8.5)	98 (10.0)	126 (7.6)		83 (8.5)	143 (8.7)	0.8	
Medicaid	434 (16.5)	149 (15.2)	285 (17.2)		160 (16.3)	272 (16.5)	0.4	
Uninsured	125 (4.7)	35 (3.6)	90 (5.4)		43 (4.4)	74 (4.5)	0.2	
Not reported	93 (3.5)	36 (3.7)	57 (3.5)		37 (3.8)	59 (3.6)	0.9	
Treatment facility								
Academic or research	740 (28.1)	270 (27.5)	470 (28.4)	.02	274 (28.0)	462 (28.0)	0.1	NA
Non–academic or research	974 (37.0)	394 (40.1)	580 (35.1)		360 (36.8)	610 (36.9)	0.3	
Not reported	922 (35.0)	318 (32.4)	604 (36.5)		345 (35.2)	580 (35.1)	0.3	
Year of diagnosis								
2010	288 (10.9)	91(9.3)	197 (11.9)	.18	106 (10.8)	180 (10.9)	0.1	NA
2011	309 (11.7)	112 (11.4)	197 (11.9)		111 (11.3)	189 (11.5)	0.4	
2012	307 (11.7)	109 (11.1)	198 (12.0)		115 (11.8)	194 (11.8)	0.0	
2013	334 (12.7)	137 (14.0)	197 (11.9)		126 (12.9)	211 (12.8)	0.3	
2014	326 (12.4)	125 (12.7)	201 (12.2)		119 (12.2)	202 (12.3)	0.3	
2015	350 (13.3)	136 (13.9)	214 (12.9)		128 (13.1)	217 (13.1)	0.2	
2016	400 (15.2)	162 (16.5)	238 (14.4)		150 (15.3)	253 (15.3)	0.1	
2017	322 (12.2)	110 (11.2)	212 (12.8)		123 (12.6)	205 (12.4)	0.7	
FIGO 2009 Stage								
IA2	347 (13.2)	151 (15.4)	196 (11.9)	.01	128 (13.1)	214 (12.9)	0.5	NA
IB1	2289 (86.8)	831 (84.6)	1458 (88.2)		851 (86.9)	1439 (87.1)	0.5	
Histologic subtype								
Squamous cell carcinoma	1352 (51.3)	476 (48.5)	876 (53.0)	.04	501 (51.2)	844 (51.1)	0.1	NA
Adenocarcinoma	1174 (44.4)	467 (47.6)	704 (42.6)		435 (44.5)	735 (44.5)	0.1	
Adenosquamous carcinoma	113 (4.3)	39 (4.0)	74 (4.5)		43 (4.4)	73 (4.4)	0.1	
Tumor grade								
1	630 (23.9)	244 (24.9)	386 (23.3)	.39	235 (24.0)	395 (23.9)	0.1	NA
2	1182 (44.8)	434 (44.2)	748 (45.2)		435 (44.5)	739 (44.8)	0.6	
3	618 (23.4)	219 (22.3)	399 (24.1)		232 (23.7)	389 (23.5)	0.4	
Not graded	206 (7.8)	85 (8.7)	121 (7.3)		77 (7.9)	129 (7.8)	0.2	
Surgical approach								
Minimally invasive surgery	1664 (63.1)	656 (66.8)	1008 (60.9)	<.001	625 (63.9)	1048 (63.4)	0.9	NA
Open surgery	853 (32.4)	248 (25.3)	605 (36.6)		309 (31.6)	529 (32.0)	0.8	
Not reported	119 (4.5)	78 (7.9)	41 (2.5)		44 (4.5)	76 (4.6)	0.3	
Postoperative metrics								
Surgical margin								
Negative (no residual disease)	2552 (97.9)	948 (97.6)	1604 (98.0)	NA	945 (97.5)	1603 (98.0)	NA	.43
Positive (with residual disease)	56 (2.2)	23 (2.4)	33 (2.0)		24 (2.5)	33 (2.0)	NA	
Not reported, No.	28	11	17		10	16	NA	
LVSI								
Negative	1864 (76.6)	702 (77.7)	1162 (75.9)	NA	703 (77.6)	1157 (75.7)	NA	.29
Positive	571 (23.5)	202 (22.4)	369 (24.1)		203 (22.4)	372 (24.3)	NA	
Not Reported	201	78	123		73	123	NA	
Postop lymph node status								
Negative	2500 (95.8)	936 (96.5)	1564 (95.4)	NA	933 (96.6)	1562 (95.3)	NA	.12
Positive	110 (4.2)	34 (3.5)	76 (4.6)		33 (3.5)	77 (4.7)	NA	
Not reported, No.	26	12	14		13	13	NA	
Readmission ≤30 d								
No	2487 (95.2)	924 (95.3)	1563 (95.1)	NA	922 (94.9)	1562 (95.2)	NA	.77
Yes	126 (4.8)	46 (4.7)	80 (4.9)		49 (5.1)	79 (4.8)	NA	
Not reported, No.	23	12	11		8	13	NA	
Adjuvant radiotherapy								
No	2242 (85.1)	813 (82.8)	1429 (86.4)	NA	823 (84.0)	1410 (85.4)	NA	.36
Yes	394 (15.0)	169 (17.2)	225 (13.6)		156 (16.0)	242 (14.7)		
Adjuvant chemotherapy								
No	2389 (90.6)	880 (89.6)	1509 (91.2)	NA	884 (90.3)	1496 (90.6)	NA	.82
Yes	247 (9.4)	102 (10.4)	145 (8.8)		95 (9.7)	156 (9.4)		
Integrated adjuvant treatment								
None	2225 (84.4)	807 (82.2)	1418 (85.7)	NA	817 (83.4)	1399 (84.7)	NA	NA
Chemoradiation	230 (8.7)	96 (9.8)	134 (8.1)		89 (9.1)	144 (8.7)		
Radiotherapy alone	164 (6.2)	73 (7.4)	91 (5.5)		67 (6.9)	98 (5.9)		
Chemotherapy alone	17 (0.6)	6 (0.6)	11 (0.7)		6 (0.6)	11 (0.7)		

Abbreviations: FIGO, International Federation of Gynecology and Obstetrics; LVSI, lymphovascular space invasion; MRH, modified radical hysterectomy; NA, not applicable; RH, radical hysterectomy; SH, simple hysterectomy.

^a^ Propensity score analysis was performed with inverse probability of treatment weighting to balance the baseline covariates between SH and RH. Specifically, a logistic model was applied to estimate each patient’s propensity to receive the SH based on age group, comorbidity score, race and ethnicity, insurance status, facility type, year of diagnosis, stage, histologic subtype, tumor grade, and surgical approach. Patients with SH were assigned a weight of (1/propensity), and patients with MRH or RH were assigned a weight of (1/[1 − propensity]). The quality of balance between the 2 groups was examined using the standardized mean difference, with a value of less than 10% considered as well-balanced. See the eAppendix in Supplement 1 for more information.

^b^ Included patients with traditional RH, MRH, or RH not otherwise specified using the 2021 National Cancer Database Participant User Data File approved and accessed in May 2024, with survival data updated through December 2023.

^c^ Patient characteristics for RH vs SH were compared using the *t* test for age at diagnosis or *χ^2^* tests for all other categorical variables.

^d^ The presence of the surgical margin, LVSI or pathologic lymph node assessment, the use of adjuvant radiotherapy or chemotherapy, and hospital readmission within 30 days after simple hysterectomy vs radical hysterectomy were compared in the propensity score balanced cohort using the weighted *χ^2^*method. The number of patients with missing data was listed in the table but were not counted in calculating proportions for the comparison.

^e^ Other includes 14 American Indian, Aleutian, Inuit, and Yupik patients; 57 patients with missing race; and 51 patients with missing ethnicity.

**Figure 1.**
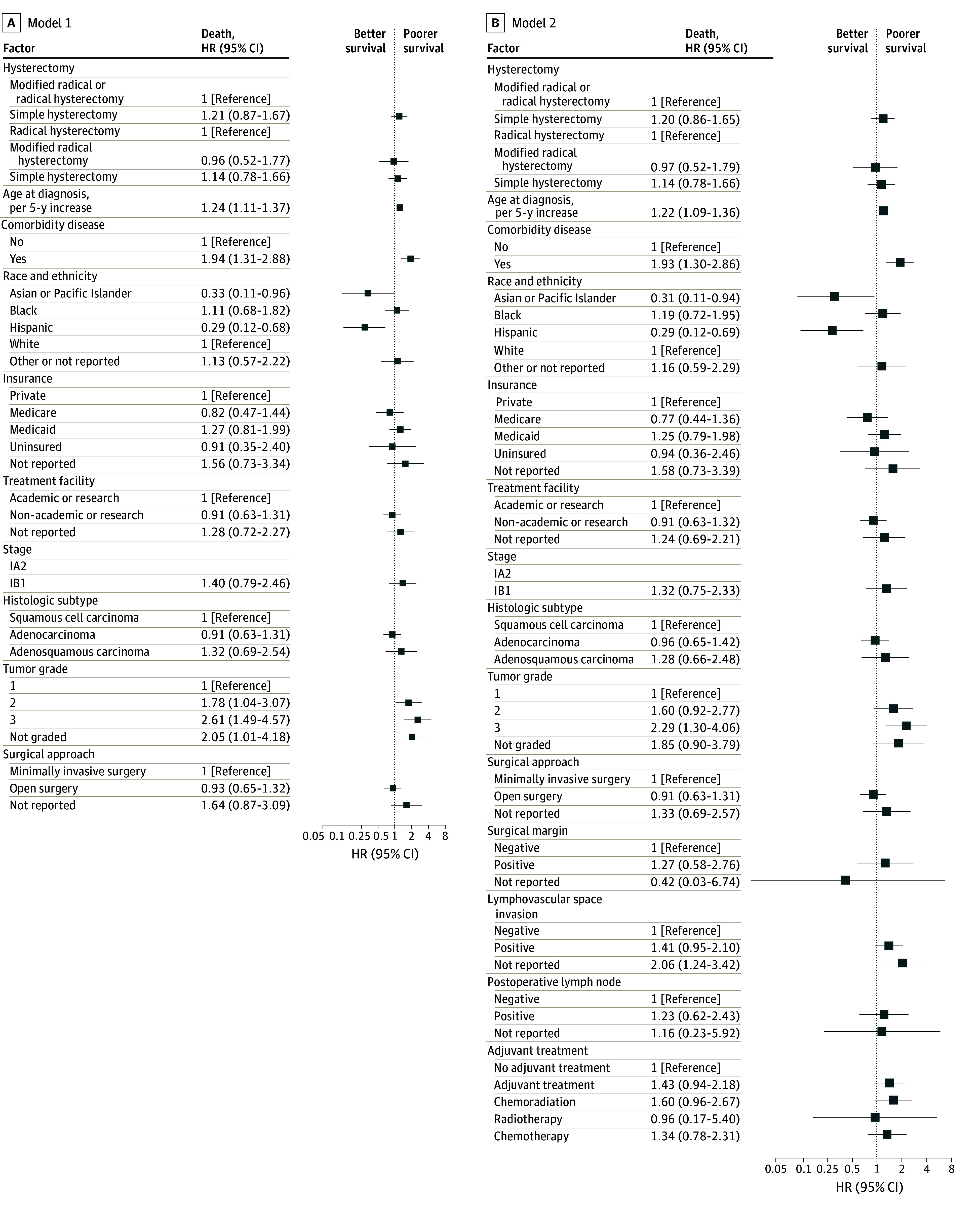
Adjusted Risk of All-Cause Death by Type of Hysterectomy and Clinical Covariates in the Original Cohort of Patients With Low-Risk Early-Stage Cervical Carcinoma Two multivariable Cox models were fitted. Model 1 (A) included the hysterectomy type and 9 baseline factors. Model 2 (B) included the model 1 variables plus 4 additional clinical factors. Other race and ethnicity included American Indian, Aleutian, Inuit, and Yupik patients, and patients with missing race or ethnicity.

Figure 1A and eTable 1 in Supplement 1 depict the results of multivariable model 1 analysis stratified by year of diagnosis and adjusted for age, comorbidity score, race and ethnicity, treatment facility, stage, histologic subtype, tumor grade, and surgical approach. There was no significant difference in the adjusted risk of all-cause death between SH and the combined MRH or RH groups (aHR, 1.21; 95% CI, 0.87-1.67; *P* = .26). There was also no difference in the pairwise comparison between SH and RH (aHR, 1.14; 95% CI, 0.78-1.66; *P* = .50) or between MRH or RH (aHR, 0.96; 95% CI, 0.52-1.77; *P* = .89). Older age at diagnosis (aHR, 1.24; 95% CI, 1.11-1.37; *P* < .001), comorbidity score of 1 or greater (aHR, 1.94; 95% CI, 1.31-2.88; *P* < .001), or grade 3 disease (aHR, 2.61; 95% CI, 1.49-4.57; *P* < .001) were associated with a higher adjusted risk of death. In contrast, Hispanic (aHR, 0.29; 95% CI, 0.12-0.68; *P* = .005) or Asian and Pacific Islander (aHR, 0.33; 95% CI, 0.11-0.96; *P* = .04) patients had a lower adjusted risk of death than non-Hispanic White patients.

Figure 1B and eTable 1 in Supplement 1 display the results of multivariable model 2, with similar risk of death for SH vs MRH or RH after extending on model 1 to also adjust for surgical margin, LVSI, pathologic LN metastasis, and administration of adjuvant radiotherapy and/or chemotherapy. Older age at diagnosis, comorbid disease, and grade 3 tumor remained poor prognostic factors. Hispanic and Asian and Pacific Islander ethnicity continued to be associated with lower risk of death in model 2 analysis.

Table 2 displays the exploratory adjusted risk of death in the original cohort of patients by subgroup. Specifically, SH vs MRH or RH had a similar adjusted risk of all-cause mortality within each subgroup categorized by age at diagnosis, comorbidity score, race and ethnicity, treatment facility, stage, histologic subtype, tumor grade, and surgical approach. There was also no evidence of an interaction between type of hysterectomy and any of these covariates (ie, *P* > .05 for each interaction test).

**Table 2.** Subgroup Analysis Displaying the Adjusted Risk of Death in the Original Cohort of Patients With Low-Risk Cervical Carcinoma Following Hysterectomy in the National Cancer Database

Characteristic	SH vs MRH or RH, adjusted HR (95% CI)^a^	*P* value	*P* for interaction
Age at diagnosis, y			
<40	1.49 (0.76-2.91)	.24	.79
40-49	1.16 (0.60-2.22)	.67	
≥50	1.13 (0.73-1.77)	.58	
Comorbidity score			
0	1.16 (0.80-1.68)	.44	.66
≥1	1.38 (0.72-2.64)	.34	
Race and ethnicity			
Asian or Pacific Islander	0.93 (0.12-7.44)	.95	.92
Black	1.26 (0.52-3.07)	.61	
Hispanic	0.59 (0.09-3.82)	.58	
White	1.22 (0.84-1.77)	.30	
Other^b^	1.73 (0.48-6.24)	.40	
Facility type			
Academic or research	1.19 (0.69-2.07)	.53	.81
Non–academic or research	1.09 (0.66-1.79)	.74	
FIGO 2009 Stage			
IA2	1.64 (0.56-4.78)	.36	.56
IB1	1.17 (0.84-1.65)	.36	
Histologic subtype			
Squamous cell carcinoma	1.32 (0.86-2.02)	.21	.72
Adenocarcinoma	1.16 (0.68-1.96)	.60	
Adenosquamous carcinoma	0.76 (0.21-2.80)	.69	
Tumor grade			
1	1.45 (0.59-3.55)	.42	.97
2	1.24 (0.76-2.01)	.40	
3	1.15 (0.67-1.98)	.61	
Not graded	1.06 (0.35-3.17)	.92	
Surgical approach			
Minimally invasive surgery	1.32 (0.87-2.00)	.19	.43
Open surgery	0.99 (0.55-1.78)	.98	

Abbreviations: FIGO, International Federation of Gynecology and Obstetrics; HR, hazard ratio; MRH, modified radical; RH, radical hysterectomy; SH, simple hysterectomy.

^a^ Subgroup analysis was conducted by extending multivariate Cox model 1 with an interaction term. Adjusted HRs and 95% CIs for risk of death in patients with SH vs RH was estimated in each subgroup, with the *P* value corresponding to the test for difference between SH and MRH or RH, and *P* for interaction values indicating the interaction test for heterogeneity of adjusted HRs by subgroup. Patients with missing value were not included for subgroup analysis and interaction test.

^b^ Other includes 14 American Indian, Aleutian, Inuit, and Yupik patients; 57 patients with missing race; and 51 with missing ethnicity.

**Figure 2.**
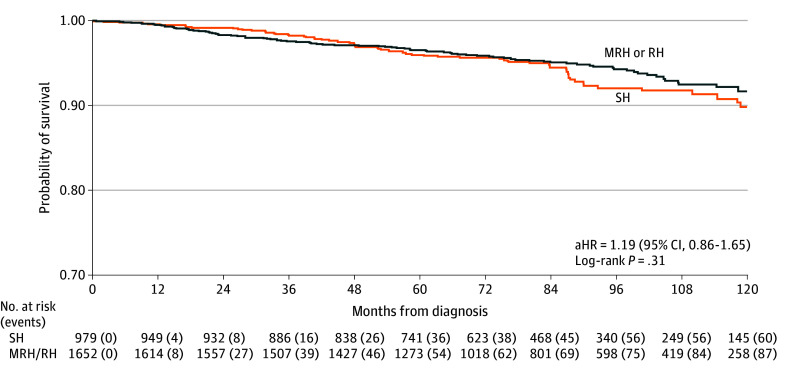
Adjusted Survival Estimates Adjusted survival was estimated using weighted Kaplan-Meier method in the propensity-score balanced cohort after applying inverse probability of treatment weighting. Adjusted hazard ratios (aHRs) for risk of death in simple hysterectomy (SH) vs modified radical hysterectomy (MRH) or radical hysterectomy (RH) were estimated using a weighted Cox model, with 95% CIs calculated using a robust sandwich covariance and inserted within the survival distribution plot.

In the propensity score–balanced cohort, adjusted risk of death was similar between SH and MRH or RH groups (aHR, 1.19; 95% CI, 0.86 to 1.65; *P* = .31) (Figure 2). Similar adjusted survival was seen in the balanced cohort following SH vs MRH or RH with consistent 3-year (98.3%; 95% CI, 97.2%-99.0% vs 97.6%; 95% CI, 96.6%-98.2%), 5-year (95.9%; 95% CI, 94.3%-97.1% vs 96.5%; 95% CI, 95.5%-97.3%), 7-year (94.5%; 95% CI, 92.5%-95.9% vs 95.1%; 95% CI, 93.7%-96.1%), and 10-year (89.8%; 95% CI86.3%-92.5% vs 91.7%; 95% CI, 89.4%-93.4%) survival rates. For example, the 7-year survival rate had a difference of −0.62% (90% CI, −2.34% to 1.10%), suggesting that the difference in survival rate between SH and MRH or RH is unlikely to exceed 2.34%, a margin consider to be clinically insignificant. The sensitivity analysis found similar 10-year RMST between SH and MRH or RH (RMST difference, −1.33; 95% CI, −3.69 to 1.03; *P* = .27), and pairwise comparisons between SH vs RH (RMST difference, −1.22; 95% CI, −3.87 to 1.43; *P* = .37), SH vs MRH (RMST difference, 0.10; 95% CI, −3.66 to 3.87; *P* = .96), and MRH vs RH (RMST difference, −1.32; 95% CI, −4.67 to 2.03; *P* = .44).

**Figure 3.**
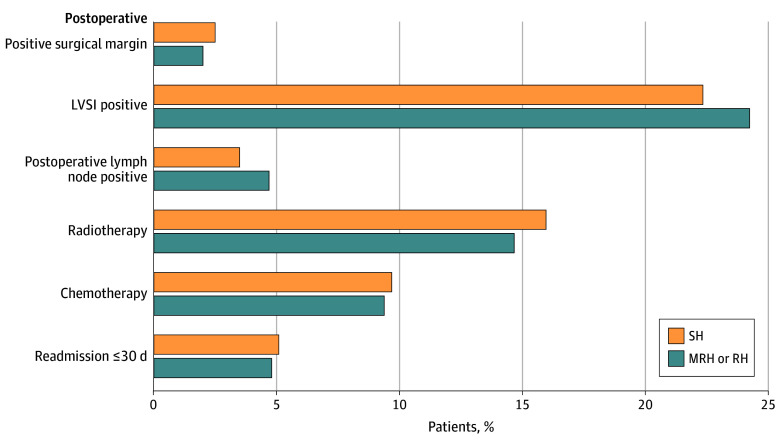
Postoperative Metrics Following Simple Hysterectomy (SH) and Modified Radical Hysterectomy (MRH) or Radical Hysterectomy (RH) Proportions between the SH and MRH or RH groups were propensity score balanced and compared using weighted χ^2^ tests. LVSI indicates lymphovascular space invasion.

Figure 3 and eTable 2 in Supplement 1 illustrate the results of our additional secondary objective examining postoperative metrics following SH vs MRH or RH. There were similar rates of positive surgical margin (24 of 969 participants [2.5%] vs 33 of 1636 participants [2.0%]; *P* = .43), presence of LVSI (203 of 906 participants [22.4%] vs 372 of 1529 participants [24.3%]; *P* = .29) or pathologic LN metastasis (33 of 967 participants [3.5%] vs 77 of 1639 participants [4.7%]; *P* = .12), and 30-day hospital readmission rate (49 of 971 participants [5.1%] vs 79 of 1640 participants [4.8%]; *P* = .77) following SH vs MRH or RH in patients propensity score balanced for the baseline clinical covariates. Utilization of radiotherapy (156 of 979 participants [16.0%] vs 242 of 1652 participants [14.7%]; *P* = .36), and administration of chemotherapy (95 of 979 participants [9.7%] vs 156 of 1652 participants [9.4%]; *P* = .82) were also similar following SH vs MRH or RH.

## Discussion

This observational cohort study of the NCDB found that patients with cervical carcinoma in Commission on Cancer–accredited facilities with FIGO 2009 stage IA2 or IB1 tumors, lesions measuring 2 cm or less, and stromal invasion less than 10 mm had similar survival following SH vs MRH or RH, providing additional support for the use of conservative surgery. Similarly, less radical surgery did not come at the expense of postoperative metrics including surgical findings, 30-day readmissions, or administration of adjuvant radiation or chemotherapy. Our study complements findings from the SHAPE trial^16^ documenting similar 3-, 5-, 7- and 10-year survival following SH vs MRH or RH in both academic or research and non–academic or research Commission on Cancer–accredited health systems. Although SHAPE discusses comparisons between SH vs RH, the RH group included patients who underwent type II RH or MRH. Herein, we also performed a sensitivity analysis with pairwise comparisons between cases coded as SH vs MRH, SH vs RH, and MRH vs RH, showing similar 10-year RMST.

Our study focused on centers within the US, whereas SHAPE included patients from the US, Western Europe, South Korea, and Canada. Our study reported similar positive surgical margin rates between SH vs MRH or RH (2.5% vs 2.0%) compared with the SHAPE trial (2.4% vs 2.7%), respectively.^16^ The rates of pathologic LN metastasis were 4.2% in our study and less than 4% in the SHAPE trial.^16^ As compared with SHAPE, our study included higher proportions with grade 3 disease (23.4% vs 14%) or positive LVSI (23.5% vs 12.9%).^16^ We cannot exclude the possibility that the similar short- and long-term survival following SH vs MRH or RH in our patients with higher risk than those included in SHAPE was attributable, at least in part, to the higher utilization of any adjuvant treatment in our study (17.8% following SH vs 14.3% following MRH or RH) compared with the SHAPE trial (9.2% following SH vs 8.4% following MRH or RH).^23^ The increased utilization of postoperative adjuvant treatment in our study followed the Peters criteria (positive nodes, parametrial involvement, or positive surgical margin) and Sedlis criteria (tumor size, stromal invasion, and LVSI).^24,25^

Previously, Sia et al^23^ used NCDB to evaluate patients who received a diagnosis of stage IA2 and IB1 cervical carcinoma less than 2 cm in size between 2004 and 2015, demonstrating a 55% increased risk for death for women with stage IB1 cancers following SH vs RH. Our study selectively included patients with lymphadenectomy, whereas Sia et al^23^ included patients who did not undergo nodal evaluation (32.9% following SH vs 4.7% following RH). Additionally, the survival analysis by Sia et al^23^ adjusted for performance of lymphadenectomy and need for adjuvant therapy, whereas our survival analysis adjusted for numerous prognostic covariates in patients with low-risk disease. In contrast, Tseng et al^26^ used the Surveillance, Epidemiology and End Results (SEER) database to show similar 10-year survival in patients who received a diagnosis between 1998 and 2012 of stage IB1 cervical carcinoma 2 cm or less in size who underwent nodal evaluation and less vs more radical surgery.

The current study also assessed the surgical approach. The trial by Ramirez et al^27^ found MIS for women with stage IA1 (LVSI), IA2, or IB1 cervical cancer was associated with a 6-fold increased risk for death compared with laparotomy. Furthermore, a study using NCDB and SEER showed similar findings.^28^ Nevertheless, debate remains whether these findings apply for those with tumors less than 2 cm given Ramirez et al^27^ was not powered to differentiate outcomes between route of surgery for those with lower-risk disease. Other studies show conflicting data for MIS for low-risk cervical carcinoma.^23,28,29,30,31,32,33,34,35,36^ Our study documented similar survival in SH vs MRH or RH performed using MIS vs open surgery. Nevertheless, our observational findings must be interpreted cautiously. Wang et al^37^ reported better short-term outcomes and similar 5-year survival between groups. We await the results of the randomized phase III clinical trials, Falconer et al^38^ and Bixel et al^39^ to provide clarity on route of surgery for early-stage cervical cancer.

### Limitations

Major limitations of our study include the inability to perform central surgicopathologic review of cases or evaluate details regarding treatments or recurrence rates. Our study design also inherently entails possibilities for selection bias, confounding, and loss to follow-up. We were also unable to explain the utilization rates of SH between 2010 and 2017, examine data on late complications (especially bladder complications), time to disease failure and sites of failure, or investigate quality of life. However, Covens et al^40^ showed that less radical surgery was associated with improved quality of life and less concern over cancer recurrence. Ferguson et al^41^ reported on sexual health and quality of life in SHAPE. The radicality of MRH vs RH varies by the disease-spread and infiltration, body habitus of the patient, and the surgeon. It is also unclear how accurately type II and III hysterectomy are captured in the NCDB. Findings from our cohort study, however, were consistent with the commentary by Nguyen et al^42^ and results from the systemic review by Wu et al.^43^

## Conclusions

Our cohort study evaluated 2636 carefully selected patients with low-risk, early-stage cervical carcinoma between 2010 and 2017, documenting similar 3-, 5-, 7-, and 10- year survival regardless of type of hysterectomy. Importantly, postoperative metrics including surgical findings, 30-day readmissions, and administration of adjuvant radiation or chemotherapy were also similar following SH vs MRH or RH. These data complement the results from SHAPE supporting the use and safety of conservative surgery in patients with low-risk, early-stage cervical carcinoma at Commission on Cancer–accredited facilities. These findings must be interpreted cautiously given our study design and higher rates of grade 3 disease, LVSI, and utilization of adjuvant therapy in our study. The similar survival irrespective of route of surgery (MIS vs open surgery) was an exploratory objective exclusively in patients at low risk and results should not be generalized. Our large observational study adds long-term survival to the mounting data supporting the use and safety of conservative surgery for low-risk, early-stage cervical carcinoma.

## Notes

Supplement 1. **eFigure.** Study Schema Displaying Patient Inclusions and Exclusions **eTable 1.** Adjusted Risk of All-Cause Death by Type Of Hysterectomy and Clinical Covariates in the Original Cohort of patients With Low-Risk Cervical Carcinoma in the National Cancer Database Using Two Multivariable Modeling Strategies **eTable 2.** Proportion With a Positive Surgical Margin, Lymphovascular Space Invasion (LVSI) Positive Disease, Pathologic Lymph Node (LN) Metastasis, Received Radiotherapy, Utilized Chemotherapy or Were Readmitted to the Hospital ≤30 Days in Propensity Score Balanced Patients With Low-Risk Cervical Carcinoma Following Simple Hysterectomy (SH) Compared With Modified Radical or Radical Hysterectomy (MRH/RH) **eAppendix.** Additional Information for Table 1

Supplement 2.
Data Sharing Statement
