# Supplementary material for: Long-Term Survival in Patients With Low-Risk Cervical Cancer After Simple, Modified, or Radical Hysterectomy
Source: JAMA Netw Open. 2025 May 15;8(5):e2510717. doi: 10.1001/jamanetworkopen.2025.10717 (PMC12082373; doi:10.1001/jamanetworkopen.2025.10717)
Supplement: Supplement 1. — eFigure. Study Schema Displaying Patient Inclusions and Exclusions eTable 1. Adjusted Risk of All-Cause Death by Type Of Hysterectomy and Clinical Covariates in the Original Cohort of patients With Low-Risk Cervical Carcinoma in the National Cancer Database Using Two Multivariable Modeling Strategies eTable 2. Proportion With a Positive Surgical Margin, Lymphovascular Space Invasion (LVSI) Positive Disease, Pathologic Lymph Node (LN) Metastasis, Received Radiotherapy, Utilized Chemotherapy or Were Readmitted to the Hospital ≤30 Days in Propensity Score Balanced Patients With Low-Risk Cervical Carcinoma Following Simple Hysterectomy (SH) Compared With Modified Radical or Radical Hysterectomy (MRH/RH) eAppendix. Additional Information for Table 1 [file jamanetwopen-e2510717-s001.pdf]

Supplemental Online Content

Tarney, CM, Tian C, Randall LM, et al. Long-term survival in patients with low-risk cervical cancer after simple, modified, or radical hysterectomy. *JAMA Netw. Open*. 2025;8(5):e2510717. doi:10.1001/jamanetworkopen.2025.10717

eFigure. Study Schema Displaying Patient Inclusions and Exclusions

eTable 1. Adjusted Risk of All-Cause Death by Type Of Hysterectomy and Clinical Covariates in the Original Cohort of patients With Low-Risk Cervical Carcinoma in the National Cancer Database Using Two Multivariable Modeling Strategies

eTable 2. Proportion With A Positive Surgical Margin, Lymphovascular Space Invasion (LVSI) Positive Disease, Pathologic Lymph Node (LN) Metastasis, Received Radiotherapy, Utilized Chemotherapy or Were Readmitted to the Hospital ≤30 Days in Propensity Score Balanced Patients With Low-Risk Cervical Carcinoma Following Simple Hysterectomy (SH) Compared With Modified Radical or Radical Hysterectomy (MRH/RH)

eAppendix. Additional Information for Table 1

This supplemental material has been provided by the authors to give readers additional information about their work.

© 2025 Tarney CM et al. *JAMA Network Open*.

eFigure. Study Schema Displaying Patient Inclusions and Exclusions

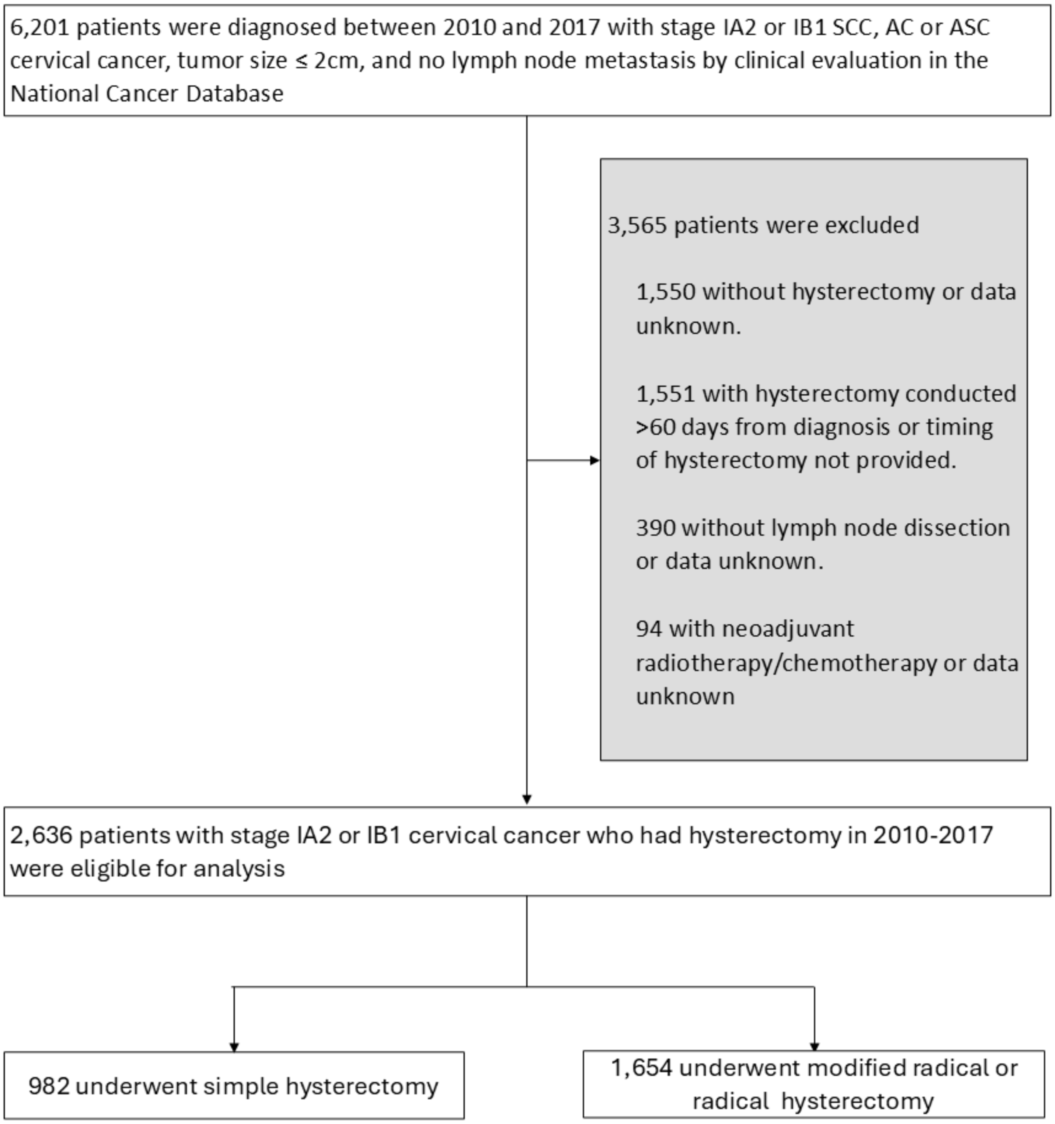

© 2025 Tarney CM et al. *JAMA Network Open*.

eTable 1. Adjusted risk of all-cause death by type of hysterectomy and clinical covariates in the original cohort of patients with low-risk cervical carcinoma in the National Cancer Database using two multivariable modeling strategies.

	Multivariable Model 1 [c]		Multivariable Model 2 [d]	
	Adjusted HR (95% CI)	*p*-value	Adjusted HR (95% CI)	*p*-value
Hysterectomy Two Categories [a]				
MRH/RH with unspecified MRH/RH	Reference		Reference	
SH	1.21 (0.87 – 1.67)	0.26	1.20 (0.86 – 1.65)	0.28
Hysterectomy Three Categories [b]				
RH	Reference		Reference	
MRH	*0.96 (0.52 – 1.77)*	*0.89*	*0.97 (0.52 – 1.79)*	*0.92*
SH	*1.14 (0.78 – 1.66)*	*0.50*	*1.14 (0.78 – 1.66)*	*0.50*
Clinical Covariates				
Age at Diagnosis				
Increasing each 5 years	1.24 (1.11 – 1.37)	<0.001	1.22 (1.09 – 1.36)	<0.001
Comorbidity Score				
0	Reference		Reference	
≥1	1.94 (1.31 – 2.88)	<0.001	1.93 (1.30 – 2.86)	0.001
Race/Ethnicity				
White	Reference		Reference	
Asian/Pacific Islander	0.33 (0.11 – 0.96)	0.04	0.31 (0.11 – 0.94)	0.04
Black	1.11 (0.68 – 1.82)	0.67	1.19 (0.72 – 1.95)	0.50
Hispanic	0.29 (0.12 – 0.68)	0.005	0.29 (0.12 – 0.69)	0.005
Other/Not Reported	1.13 (0.57 – 2.22)	0.73	1.16 (0.59 – 2.29)	0.67
Insurance Status				
Private	Reference		Reference	
Medicare	0.82 (0.47 – 1.44)	0.49	0.77 (0.44 – 1.36)	0.37
Medicaid	1.27 (0.81 – 1.99)	0.31	1.25 (0.79 – 1.98)	0.34
Uninsured	0.91 (0.35 – 2.40)	0.85	0.94 (0.36 – 2.46)	0.89
Not Reported	1.56 (0.73 – 3.34)	0.25	1.58 (0.73 – 3.39)	0.24
Treatment Facility				
Academic/Research	Reference		Reference	
Non-Academic/Research	0.91 (0.63 – 1.31)	0.61	0.91 (0.63 – 1.32)	0.63
Not Reported	1.28 (0.72 – 2.27)	0.40	1.24 (0.69 – 2.21)	0.47
FIGO 2009 Stage				
IA2	Reference		Reference	
IB1	1.40 (0.79 – 2.46)	0.25	1.32 (0.75 – 2.33)	0.34
Histology				
Squamous Cell Carcinoma	Reference		Reference	
Adenocarcinoma	0.91 (0.63 – 1.31)	0.61	0.96 (0.65 – 1.42)	0.85
Adenosquamous Carcinoma	1.32 (0.69 – 2.54)	0.41	1.28 (0.66 – 2.48)	0.46
Tumor Grade				
1	Reference		Reference	
2	1.78 (1.04 – 3.07)	0.04	1.60 (0.92 – 2.77)	0.10
3	2.61 (1.49 – 4.57)	<0.001	2.29 (1.30 – 4.06)	0.004
Not Graded	2.05 (1.01 – 4.18)	0.05	1.85 (0.90 – 3.79)	0.09
Surgical Approach				

© 2025 Tarney CM et al. *JAMA Network Open*.

Minimally Invasive Surgery	Reference		Reference	
Open Surgery	0.93 (0.65 – 1.32)	0.67	0.91 (0.63 – 1.31)	0.61
Not Reported	1.64 (0.87 – 3.09)	0.13	1.33 (0.69 – 2.57)	0.39
Surgical Margin				
Negative (No Residual Disease)			Reference	
Positive (with Residual Disease)			1.27 (0.58 – 2.76)	0.56
Not Reported			0.42 (0.03 – 6.74)	0.54
Lymphovascular Space Invasion				
Negative			Reference	
Positive			1.41 (0.95 – 2.10)	0.09
Not Reported			2.06 (1.24 – 3.42)	0.005
Post-Op Lymph Node Status				
Negative			Reference	
Positive			1.23 (0.62 – 2.43)	0.56
Not Reported			1.16 (0.23 – 5.92)	0.86
Adjuvant Treatment				
None			Reference	
Adjuvant Treatment			1.43 (0.94 – 2.18)	0.10
Chemoradiation			*1.60 (0.96 – 2.67)*	*0.07*
Radiotherapy alone			*0.96 (0.17 – 5.40)*	*0.96*
Chemotherapy alone			*1.34 (0.78 – 2.31)*	*0.28*

Abbreviations: SH: simple hysterectomy, MRH: modified radical, RH: radical hysterectomy, HR: hazard ratio, CI: confidence interval.

a. Adjusted risk of all-cause death was estimated for patients undergoing a SH compared with MRH/RH including MRH, traditional RH or unspecified MRH/RH.
b. Adjusted risk of all-cause death was estimated for either SH or MRH vs. RH after excluding patients who underwent an unspecified MRH or RH.
c. Multivariable Model 1 analysis of clinical factors associated with survival was conducted using a stratified Cox model (stratified by year of diagnosis) with adjusted HR and 95% CI for risk of death estimated after controlling for hysterectomy type, age, comorbidity score, race/ethnicity, stage, histology, tumor grade, and surgical approach.
d. Multivariable Model 2 analysis of clinical factors associated with survival was conducted using a stratified Cox model (stratified by year of diagnosis) with adjusted HR and 95% CI for risk of death estimated after controlling for the hysterectomy type, age, comorbidity score, race/ethnicity, stage, histology, tumor grade, and surgical approach plus surgical margin, LVSI, pathologic lymph node assessment, and adjuvant treatment.

© 2025 Tarney CM et al. *JAMA Network Open*.

eTable 2. Proportion with a positive surgical margin, lymphovascular space invasion (LVSI) positive disease, pathologic lymph node (LN) metastasis, received radiotherapy, utilized chemotherapy or were readmitted to the hospital ≤30 days in propensity score balanced patients with low-risk cervical carcinoma following simple hysterectomy (SH) compared with modified radical or radical hysterectomy (MRH/RH).

	Proportion (%)	
	SH	MRH/RH
Positive Surgical Margin	2.5	2
LVSI Positive	22.4	24.3
Post-Op Lymph Node Positive	3.5	4.7
Radiotherapy	16	14.7
Chemotherapy	9.7	9.4
Readmission within 30 Days	5.1	4.8

Proportions between the SH and MRH/RH groups were compared using weighted Chi-square test.

© 2025 Tarney CM et al. *JAMA Network Open*.

eAppendix. Additional Information for Table 1.

The race and ethnicity variables provided by the National Cancer Database were crossed to generate the race and ethnicity groups, including non-Hispanic White patients (hereafter referred to as White patients), non-Hispanic Black patients (hereafter referred to as Black patients), Hispanic patients (including any or missing race), Asian or Pacific Islander patients, and patients with other or not reported race and ethnicity (httpw://www.facs.org).

Insurance status indicated the primary insurance carrier at the time of diagnosis.

Histologic subtypes were classified using the *International Classification of Disease for Oncology, third edition* (*ICD-O-3*). Squamous cell carcinoma was identified using codes 8052, 8070-8078; Adenocarcinoma using codes 8140-8141, 8143, 8147, 8255, 8260-8263, 8310, 8323, 8380, 8384, 8430 and 8480-8482; and adenosquamous carcinoma using codes 8560, 8570-8575.

The number of patients with missing data in surgical margin, LVSI, pathologic lymph node assessment or readmission within 30 days was listed in the table but were not counted in calculating proportions for the comparison between MRH or RH vs SH.

Comorbidity score was coded by the National Cancer Database using the Charlson-Deyo index system (PMID: 1607900) and for this study was categorized as 0 or 1 or greater (httpw://www.facs.org). This scoring system considers the following conditions to be a level 1: myocardial infarction, congestive heart failure, peripheral vascular disease, cerebrovascular disease, dementia, chronic pulmonary disease, rheumatologic disease, peptic ulcer disease, mild liver disease, and diabetes. Level 2 consists of diabetes with chronic complications, hemiplegia or paraplegia, and kidney disease. Level 3 consists of moderate or severe liver disease. Level 6 consists of acquired immunodeficiency syndrome. A 0 score indicates the patient had none of the conditions in the Charlson-Deyo index mapping levels; however, the patients could have other comorbid conditions.

© 2025 Tarney CM et al. *JAMA Network Open*.
