# Supplementary material for: Long-Term Survival in Patients With Low-Risk Cervical Cancer After Simple, Modified, or Radical Hysterectomy
Source: JAMA Netw Open. 2025 May 15;8(5):e2510717. doi: 10.1001/jamanetworkopen.2025.10717 (PMC12082373; doi:10.1001/jamanetworkopen.2025.10717)
Supplement: Supplement 2. — Data Sharing Statement [file jamanetwopen-e2510717-s002.pdf]

Data Sharing Statement

Tarney. Long-Term Survival in Low-Risk Patients With Cervical Cancer After Simple, Modified, or Radical Hysterectomy. *JAMA Netw Open*. Published May 15, 2025. doi:10.1001/jamanetworkopen.2025.10717

Data
Data available: No

Additional Information
Explanation for why data not available: The dataset utilized to perform this investigation was obtained from the Commission on Cancer's National Cancer Database (NCDB) for researchers who meet the criteria for access to data. The NCDB Participant Use Data File (PUF) is a Health Insurance Portability and Accountability Act (HIPAA)-compliant data file containing cases submitted to the Commission on Cancer's (CoC) NCDB. The PUF contains de-identified patient level data that do not identify hospitals, health care providers, or patients as agreed to in the Business Associate Agreement that each CoC-accredited program has signed with the American College of Surgeons. Restrictions apply to the dataset: The analyses presented in this article utilized a dataset that is not readily available as it was acquired through a restricted access approval process from the National Cancer Database with a data use agreement that prohibits sharing (https://www.facs.org/quality-programs/cancer-programs/national-cancer-database). Questions regarding the NCDB PUF or the application process for a PUF may be directed to NCDB technical staff at NCDB_PUF@facs.org. Requests to access the dataset should be directed to the following instructions for applications to the NCDB (https://www.facs.org/media/xtvknrsu/2020-puf-instructions-to-potential-applicants.pdf).
